# Zoonotic *Rickettsia* Species in Small Ruminant Ticks From Tunisia

**DOI:** 10.3389/fvets.2021.676896

**Published:** 2021-05-26

**Authors:** Hanène Belkahia, Rachid Selmi, Sayed Zamiti, Monia Daaloul-Jedidi, Lilia Messadi, Mourad Ben Said

**Affiliations:** ^1^Service de Microbiologie et Immunologie, Ecole Nationale de Médecine Vétérinaire, University of Manouba, Sidi Thabet, Tunisia; ^2^Ministère de la Défense Nationale, Direction Générale de la Santé Militaire, Service Vétérinaire, Tunis, Tunisia; ^3^Département des Sciences Fondamentales, Institut Supérieur de Biotechnologie de Sidi Thabet, University of Manouba, Sidi Thabet, Tunisia

**Keywords:** *Rickettsia* species, *Rhipicephalus* ticks, molecular survey, genotyping, phylogenetic analysis, Tunisia

## Abstract

Tick-borne rickettsioses present a significant public health threat among emerging tick-borne diseases. In Tunisia, little is known about tick-borne *Rickettsia* pathogens. Therefore, the aim of this study was to investigate the presence of *Rickettsia* species in small ruminant ticks from Tunisia. Adult ticks (*n* = 694) were collected from goats and sheep in northern Tunisia. Obtained ticks were identified as *Rhipicephalus turanicus* (*n* = 434) and *Rhipicephalus sanguineus* sensu lato (*n* = 260). Selected ticks (*n* = 666) were screened for the presence of *Rickettsia* spp. by PCR targeting a partial sequence of the *ompB* gene followed by sequence analysis. Rickettsial DNA was detected in 122 (18.3%) tested tick samples. The infection rates in *Rh. turanicus* and *Rh. sanguineus* s.l. ticks were 23.4 and 9.5%, respectively. The overall prevalence of rickettsial DNA was markedly higher in ticks collected from goats (23.2%) compared to those infesting sheep (7.9%). The detection of rickettsial DNA was significantly higher in ticks from the governorate of Beja (39.0%) than those from the governorate of Bizerte (13.9%). Two additional genes, the outer membrane protein A gene (*ompA*) and the citrate synthase gene (*gltA*), were also targeted for further characterization of the detected *Rickettsia* species. Genotyping and phylogenetic analysis based on partial sequences (*n* = 106) of the three different genes revealed that positive ticks are infected with different isolates of two Spotted Fever Group (SFG) *Rickettsia*, namely, *Rickettsia massiliae* and *Rickettsia monacensis*, closely related to those infecting camels and associated ticks from Tunisia, and humans and small ruminant ticks from neighboring countries like Italy, France, and Spain.

## Introduction

*Rickettsia* species (family Rickettsiaceae; order Rickettsiales) are included into four groups: the spotted fever group (SFG) rickettsiae, the typhus group, the *Rickettsia bellii* group, and the *Rickettsia canadensis* group (1). These pathogens infected several domesticated and wild vertebrate hosts through hematophagous arthropod vectors bites (mainly ticks, fleas, and mites). Besides, tick-borne rickettsioses are considered as one of the most virulent zoonotic diseases affecting humans especially in African countries (2).

Spotted fever group rickettsioses (SFG) are actually considered as emerging and reemerging diseases affecting animals worldwide. They are caused by the pathogenic and zoonotic spotted fever *Rickettsia* bacteria mainly transmitted by ticks. Humans may be accidently infected especially in tropical areas (1, 2).

In Tunisia, several SFG *Rickettsia* species have been previously reported, as *Rickettsia conorii*, that was described for the first time in humans since 1910 (3), and, recently, by Znazen et al. (4) and Khrouf et al. (5). In addition, *R. conorii* subsp. *israelensis* was identified in one human and tick specimens of *Rhipicephalus sanguineus* s.l. complex collected from dogs (4, 6). Furthermore, *R. aeschlimannii, R. helvetica*, and *R. africae* were reported from camels' blood samples and infesting *Hyalomma* tick tissues in southern and central Tunisia (7, 8). DNA of *R. helvetica* was also identified in questing *Ixodes ricinus* ticks (9).

*Rickettsia massiliae* and *Rickettsia monacensis*, belonging to the SFG rickettsiae, are widely identified among animals, humans, and arthropod vectors (1). *Rickettsia massiliae* was firstly isolated in France from *Rhipicephalus turanicus* tick (10). Since then, this pathogen has been transmitted by and/or isolated from *Rhipicephalus* ticks like *Rh. turanicus, Rh. sanguineus* sensu lato (s.l.), *Rh. bursa*, and *Rh. pusillus* collected from domestic and wild animals such as cattle, goats, horses, dogs, cats, hedgehogs, red foxes, and hares in different worldwide countries (11–16). In Tunisia, *R. massiliae* was previously detected in *Rh. sanguineus* s.l. ticks collected from dogs (6), in peripheral blood of camels (8), and in skin biopsy of one patient (5). Interestingly, this bacterium is recognized as pathogenic in human and may be clinically expressed as a febrile illness with maculopapular rash, fever, night sweats, headache, and necrotic eschar at the tick bite site (17, 18).

*Rickettsia monacensis* was earlier detected in *I. ricinus* ticks from several European countries like Italy, Spain, Romania, Bulgaria, Hungary, and Serbia (1, 12). In our country, the first identification of *R. monacensis* was also reported in *I. ricinus* ticks by Sfar et al. (9). Additionally, this human-pathogenic species was recently detected not only in Tunisian camels but also in associated *H. impeltatum* ticks removed from uninfected animals (8). This bacterium causes from moderate to severe infections in humans including fever, rash on palms and soles, and inoculation eschar (19, 20). To better understand the epidemiology of *Rickettsia* species in Tunisia, we investigated, in the present molecular survey the occurrence of rickettsial bacteria in small ruminant ticks according to potential risk factors. Molecular characterization and phylogenetic analysis of revealed *Rickettsia* spp. isolates were also performed by using three different gene fragments.

## Materials and Methods

### Study Area Description

**Figure 1:**
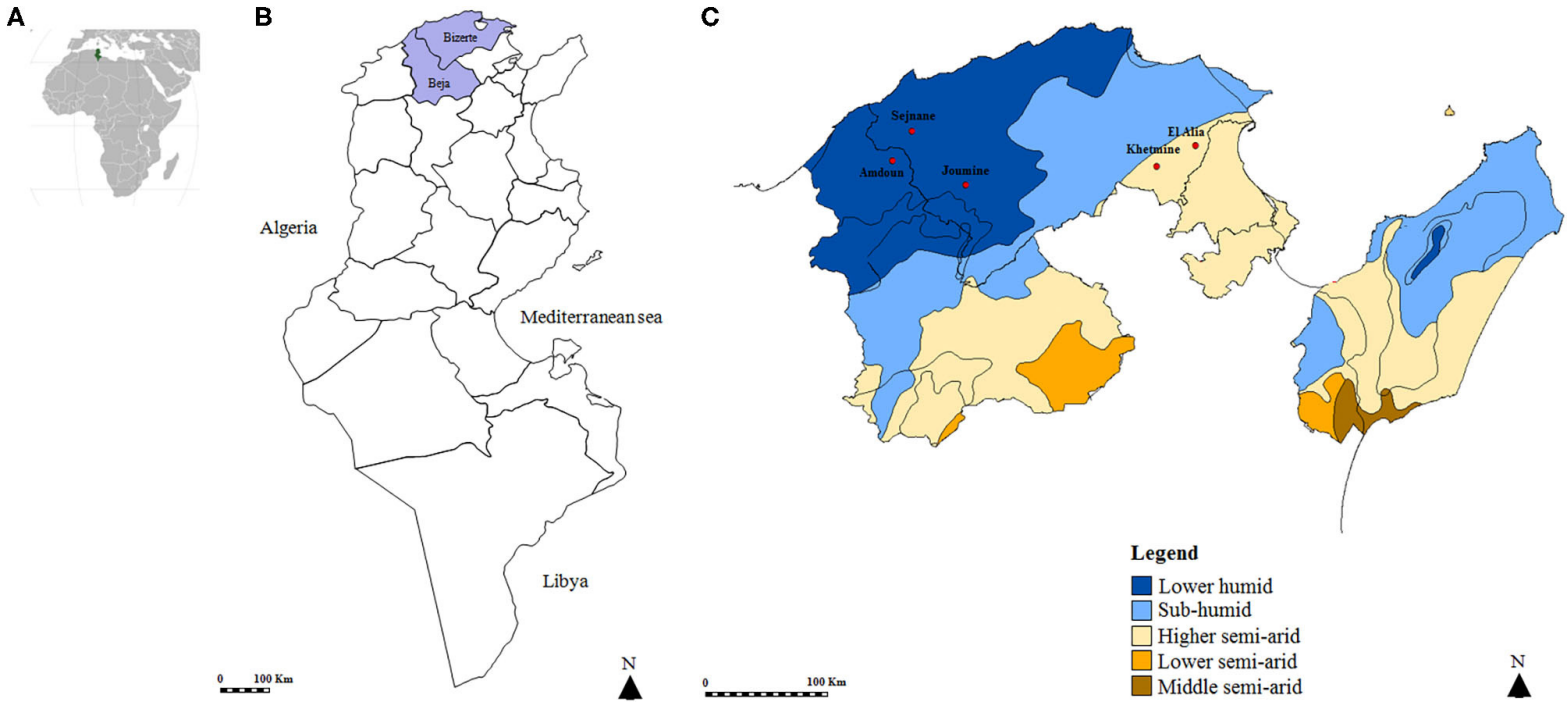
Map of the Tunisian studied regions. **(A)** Geographical position of Tunisia in the African continent. **(B)** Map of Tunisia showing investigated governorates. **(C)** Position of studied localities according to bioclimatic areas.

A cross-sectional study was carried out in five localities of Northern Tunisia (Figure 1). El Alia 37°16′ N; 10°03′ E and Khetmine 37°16′ N; 9°99′ E fall in the sub-humid bioclimatic zone with an average annual rainfall of 400 mm and a mean temperature of 18.4°C while Joumine 36°92′ N; 9°38′ E, Sejnane 37°15′ N; 9°23′ E, and Amdoun 36°76′ N; 9°08′ E are characterized by humid climate with an average annual rainfall of 650 mm and a mean temperature of 14.4°C.

### Tick Collection and Identification

Ticks were collected from 303 apparently healthy goats (233 doe and 70 buck) and 160 healthy sheep (110 ewes and 50 rams). Goats were originated from 16 herds located in Sejnane (*N* = 3), El Alia (*N* = 4), and Joumine (*N* = 5) belonging to the Bizerte governorate and in Amdoun (*N* = 4, Beja governorate). Sheep derived from nine herds from El Alia (*N* = 4) and Khetmine (*N* = 5) in the governorate of Bizerte.

All partially engorged ticks were collected by using a clamp from different preferred sites of small ruminant body (ears, neck, udder, and external genitalia) and separately categorized according to the examined animal host. Obtained specimens were morphologically identified using the taxonomic key of Walker et al. (21) and then classified according to tick species, life stage, and gender. Each tick specimen was individually conserved in a tube containing 70% ethanol and stored at −20°C.

### Total DNA Extraction and Tick DNA Amplification

Each identified tick was washed with sterile water, dried, and crushed individually using an automated TissueLyser LT system (Qiagen, Hilden, Germany). Genomic DNA extraction was performed from each tick sample using the DNeasy tissue kit (Qiagen, Hilden, Germany). Obtained DNA extracts were stored at −20°C. DNA extraction efficiency was validated by PCR amplification step targeting the ribosomal RNA subunit (16S rRNA) gene using the tick-specific primers TQ16S+1F and TQ16S-2R as described by Black and Piesman (22) (Table 1).

**Table 1:** Primers used for the identification and/or genetic characterization of *Rickettsia* species infecting ticks collected in this study from small ruminants.

**Assays**	**Target genes**	**Primers**	**Sequences (5^**′**^-3^**′**^)**	**Amplicon size (bp)**	**References**
**Single PCR**^**a**^					
	16S rRNA	TQ16S+1F	CTGCTCAATGATTTTTTAAATTGCTGTGG	324	(22)
		TQ16S-2R	ACGCTGTTATCCCTAGAG		
**Nested PCR**^**b**^					
First PCR	*ompB*	rompB_OF	GTAACCGGAAGTAATCGTTTCGTAA	511	(23)
		rompB OR	GCTTTATAACCAGCTAAACCACC		
Second PCR		rompB_SFG_IF	GTTTAATACGTGCTGCTAACCAA	425	
		rompB SFG-IR	GGTTTGGCCCATATACCATAAG		
**Semi-nested PCR**^**c**^					
First PCR	*ompA*	Rr190.70p	ATGGCGAATATTTCTCCAAAA	631	(24)
		Rr190.701n	GTTCCGTTAATGGCAGCATCT		
Second PCR		Rr190.70p	ATGGCGAATATTTCTCCAAAA	532	
		Rr190.602n	AGTGCAGCATTCGCTCCCCCT		
**Single PCR**^**c**^
	*gltA*	RpCS.877p	GGGGGCCTGCTCACGGCGG	381	(25)
		RpCS.1258n	ATTGCAAAAAGTACAGTGAACA		

a *Single PCR based on the 16S rRNA gene allowing the selection of tick samples with DNA extraction efficiency*.

b *Nested PCR based on the ompB gene allowing the detection and/or characterization after sequencing of Rickettsia species*.

c *Single and semi-nested PCR based on gltA and ompA genes, respectively, allowing the characterization after sequencing of Rickettsia species*.

### Molecular Detection of *Rickettsia* spp.

In order to identify all species of the *Rickettsia* genus, tick DNA samples were subjected to nested PCR targeting a fragment (425 bp) of the rickettsial outer membrane protein B (*ompB*) gene (23) (Table 1). For further characterization, the outer membrane protein A (*ompA*) and the citrate synthase protein (*gltA*) gene fragments (532 and 381 bp, respectively) were amplified by using nested and endpoint PCR, respectively (Table 1). PCR reactions were performed in an automated DNA thermal cycler. Thermal cycling profiles were as described by Oteo et al. (24), and Regnery et al. (25), respectively.

The PCR reactions were carried out in a final volume of 50 μl composed of 0.125 U/μL of Taq DNA polymerase (Biobasic Inc., Markham, Canada), 1 × PCR buffer, 1.5 mM MgCl2, 0.2 mM of dNTP, 3 μL of genomic DNA (50–150 ng) in the first PCR and 1 μL in the second PCR (for nested PCR), 0.5 μM of the primers, and autoclaved water. PCR products were visualized using electrophoresis in 1.5% agarose gels stained with ethidium bromide and observed under UV transillumination.

### Statistical Analysis

Exact confidence intervals (CI) at the 95% level were estimated for prevalence rates according to different considered factors. A comparison of the prevalence of *Rickettsia* species in ticks according to abiotic factors (geographic location and bioclimatic conditions) and factors related to ticks (gender, age, and host origin) was carried out using the Epi Info 6 software 01 (CDC, Atlanta, USA) and the χ^2^-test. A difference is considered statistically significant when the degree of significance *p* is ≤0.05. In order to assess possible confusion between the risk factors, a Mantel–Haenszel χ^2^-test was performed.

### DNA Sequencing and Obtaining Final Sequences

A total of 106 positive PCR products obtained after *ompB, ompA*, and *gltA* PCRs were randomly selected and purified using the GF-1 Ambi Clean kit (Vivantis, USA), according to the manufacturer's instructions. Purified DNA amplicons were sequenced in both directions, using the same primers as for the single *gltA* PCR and the second PCR of each nested PCR amplification targeting *ompA* and *ompB* genes. The Big Dye Terminator cycle sequencing ready reaction kit (Applied Biosystems, Foster City, USA) and an ABI3730XL automated DNA sequencer (Macrogen Europe, Amsterdam, The Netherlands) were employed.

The chromatograms were evaluated with Chromas Lite v 2.01 (http://www.technelysium.com.au/chromas_lite.html). To obtain maximal data accuracy, sequences were determined on both forward and reverse strands. Indeed, the complementary strands of each sequenced product were manually assembled by using the DNAMAN software (Version 5.2.2; Lynnon Biosoft, Que., Canada). The primer region sequences were automatically removed and the overlapping parts were selected.

### Sequence Alignment and Phylogenetic Study

Multiple-sequence alignments and sequence similarities were calculated using the CLUSTAL W method (26). BLAST analysis was performed to assess the level of similarity with previously reported sequences (http://blast.ncbi.nlm.nih.gov/). By using the DNAMAN software, genetic distances among the operational taxonomic units were computed by the maximum composite likelihood method (27) and were used to construct neighbor-joining trees (28). Statistical support for internal branches of trees was evaluated by bootstrapping with 1,000 iterations (29).

## Results

### Tick Species Recognition

A total of 694 ticks were collected from goats (460/694, 66.3%) and sheep (234/694, 33.7%) from a higher semiarid area (374/694, 53.9%) and a low humid area (320/694, 46.1%). Almost all specimens were removed from animals located in the governorate of Bizerte (82%) while ticks collected from small ruminants in El Alia were the most numerous (43.5%) compared to those in other localities (Figure 1 and Table 2). The sex ratio of ticks collected from these animals (M/F) was 1.14. The intensity of tick infestation is estimated at 1.52 and 1.46 ticks/animal for goats and sheep, respectively. Two tick species belonging to *Rhipicephalus* genus were identified, namely, *Rh. turanicus* (434/694, 62.5%) and *Rh. sanguineus* s.l. (260/694, 37.5%) (Table 2).

**Table 2:** Molecular prevalences of *Rickettsia* spp. according to tick species, tick gender, infested host, bioclimatic zone, governorate, and locality.

**Factors**	**Number of collected ticks (%)^**a**^**	**Number of analyzed ticks (%)^**b**^**	**Positive^**c**^ (% ± C.I.^**d**^)**	***P-*value (Khi2)**
Tick species				0.000^*^ (20.02)
*Rh. turanicus*	434 (62.5)	423 (63.5)	99 (23.4 ± 0.04)	
*Rh. sanguineus* s.l.	260 (37.5)	243 (36.5)	23 (9.5 ± 0.04)	
Tick gender				0.519 (0.42)
Male	370 (53.3)	356 (53.4)	62 (17.4 ± 0.04)	
Female	324 (46.7)	310 (46.6)	60 (19.4 ± 0.04)	
Infested host				0.000^*^ (22.65)
Goats	460 (66.3)	452 (67.9)	105 (23.2 ± 0.04)	
Sheep	234 (33.7)	214 (32.1)	17 (7.9 ± 0.03)	
Bioclimatic zone				0.185 (1.76)
Higher semi-arid	374 (53.9)	357 (53.6)	72 (20.2 ± 0.04)	
Lower humid	320 (46.1)	309 (46.4)	50 (16.2 ± 0.04)	
Governorate				0.000^*^ (40.87)
Bizerte	569 (82.0)	548 (82.3)	76 (13.9 ± 0.03)	
Beja	125 (18.0)	118 (17.7)	46 (39.0 ± 0.09)	
Locality				0.000^*^ (87.96)
El Alia	302 (43.5)	289 (43.4)	71 (24.6 ± 0.05)	
Khetmine	72 (10.4)	68 (10.2)	1 (1.5 ± 0.03)	
Sejnane	137 (19.7)	133 (20.0)	4 (3.0 ± 0.03)	
Amdoun	125 (18.0)	118 (17.7)	46 (39.0 ± 0.09)	
Joumine	58 (8.4)	58 (8.7)	0 (0)	
Total	694 (100)	666 (100)	122 (18.3 ± 0.03)	

*Rh. Turanicus, Rhipicephalus turanicus, Rh. sanguineus s.l., Rhipicephalus sanguineus sensu lato*.

a *Number of collected ticks submitted to PCR performed for the confirmation of the DNA extraction efficiency*.

b *Number of included ticks for Rickettsia spp. survey selected after the confirmation of the DNA extraction efficiency*.

c *Ticks positive to Rickettsia spp. according to the total number of analyzed ticks*.

d *C.I.: 95% confidence interval*.

* *Statistically significant test*.

### Efficiency of DNA Isolation

DNA extracts were tested and validated in 666 samples (96%). No amplification products were obtained for 28 samples, reflecting a probable failure of the DNA extraction, and were thus excluded from the analysis. Thereby, a total of 666 ticks were selected from goats (452/666, 67.9%) and sheep (214/666, 32.1%) from the higher semiarid area (357/666, 53.6%) and the low humid area (309/666, 46.4%). Almost all analyzed ticks were collected from small ruminants located in the governorate of Bizerte (82.3%) while ticks collected from animals in El Alia are the most numerous (43.4%) compared to those in other localities (Figure 1 and Table 2). The sex ratio of tested ticks (M/F) was 1.15. After the validation of DNA extracts, a total of 423 *Rh. turanicus* (63.5%) and 243 *Rh. sanguineus* s.l. (36.5%) were subjected to *Rickettsia* spp. screening (Table 2).

### *Rickettsia* spp. Screening and Risk Factor Analysis

Based on *ompB* gene analysis, DNA of *Rickettsia* spp. was identified in 122 tick samples (18.3%) (Table 2). Infection among *Rh. turanicus* ticks is statistically more prevalent (23.4%) compared to *Rh. sanguineus* s.l. (9.5%) (*p* < 0.001). Ticks collected from goats were statistically more infected with *Rickettsia* spp. (23.2%) than those from sheep (7.9%) (*p* < 0.001; Table 2). Ticks removed from small ruminants located in the governorate of Beja were statistically more infected with *Rickettsia* spp. (39.0%) (*p* < 0.001) than those in the governorate of Bizerte (13.9%) (*p* < 0.001). Specimens from Amdoun (39.0%) and El Alia (24.6%) localities were more infected with *Rickettsia* spp. than those from Sejnane (3.0%), Khetmine (1.5%), and Joumine (0%) (*p* < 0.001; Table 2). In contrast, no statistically significant differences in *Rickettsia* spp. infection rates were observed according to tick gender and bioclimatic areas (*p* < 0.05, Table 2).

### Identification of *Rickettsia* Species Infecting Ticks

Two rickettsial species were identified in small ruminants' ticks, namely, *R. massiliae* and *R. monacensis* (Table 3). Based on *ompB* gene analysis, 40 PCR products (32 from *Rh. turanicus* and eight from *Rh. sanguineus* s.l.) were sequenced successfully. *Rickettsia massiliae* was identified in *Rh. turanicus* (*n* = 32, 100%) and *Rh. sanguineus* s.l. (*n* = 6, 75%). However, *R. monacensis* DNA was found in *Rh. sanguineus* s.l. (*n* = 2, 25%). Based on *ompA* and *gltA* gene analysis, PCR products were sequenced successfully from 41 and 25 positives samples, respectively. *Rickettsia massiliae* was detected in *Rh. turanicus* (*n* = 31, 100%) and *Rh. sanguineus* s.l. (*n* = 10, 100%) based on *ompA* partial sequence analysis. However, using the *gltA* gene, DNA of this bacterium was found in *Rh. turanicus* (*n* = 25, 100%) (Table 3).

**Table 3:** *Rickettsia* species identified by the sequencing of *ompB, ompA*, and *gltA* partial sequences in *Rhipicephalus* ticks.

**Tick species**	**Number**	***ompB* PCR positive (%)**	***ompB* PCR positives/sequencing**	***ompA* PCR positives/sequencing**	***gltA* PCR positives/sequencing**	***Rickettsia* spp**.
*Rh. turanicus*	423	99 (23.4 ± 0.04)	32	31	25	*R. massiliae*
			0	0	0	*R. monacensis*
*Rh. sanguineus* s.l.	243	23 (9.5 ± 0.04)	6	10	0	*R. massiliae*
			2	0	0	*R. monacensis*
Total	666	122 (18.3 ± 0.03)	40	41	25	*Rickettsia* spp.

*Rh. turanicus, Rhipicephalus turanicus; Rh. sanguineus s.l., Rhipicephalus sanguineus sensu lato*.

### Molecular Characterization and Phylogenetic Analysis

Out of 122 *Rickettsia*-positive samples, 94 gave a clear band in the correct nucleotide size of the partial genes (*ompA, ompB*, and *gltA*) in at least one of the three genotyping PCRs. Partial sequences (*n* = 106) of the three analyzed genes were deposited under GenBank accession numbers presented in Table 4. Based on all revealed sequences of the three analyzed genes, we precisely selected *Rickettsia* spp. genotypes according to infecting tick species, and they differ from each other by at least one mutation in the nucleotidic sequence.

**Table 4:** Designation and information about sequencing of *Rickettsia* spp. genotypes identified in this study.

**Gene**	***Rickettsia* sp**.	**Genotype**	**Number^**a**^**	**Potential vector**	**Location^**b**^**	**GenBank^**c**^**	**BLAST analysis**
*ompB*	*R. massiliae*	ompBRmasRs1	4	*Rh. sanguineus* s.l.	Bizerte	MN311185	100% *R. massiliae* (CP000683)
		ompBRmasRs2	2	*Rh. sanguineus* s.l.	Bizerte	MN311189	100% *R. massiliae* (KJ663751)
		ompBRmasRt1	20	*Rh. turanicus*	Bizerte and Beja	MN311191	100% *R. massiliae* (CP000683)
		ompBRmasRt2	12	*Rh. turanicus*	Bizerte and Beja	MN311211	100% *R. massiliae* (KJ663751)
	*R. monacensis*	ompBRmonRs1	1	*Rh. sanguineus* s.l.	Bizerte	MN311223	100% *R. monacensis* (EU883092)
		ompBRmonRs2	1	*Rh. sanguineus* s.l.	Bizerte	MN311224	99.4% *R. monacensis* (EU883092)
*ompA*	*R. massiliae*	ompARmasRs1	6	*Rh. sanguineus* s.l.	Bizerte	MN311225	100% *R. massiliae* (MH532237)
		ompARmasRs2	2	*Rh. sanguineus* s.l.	Bizerte	MN311229	100% *R. massiliae* (KJ663747)
		ompARmasRs3	1	*Rh. sanguineus* s.l.	Beja	MW026194	99.8% *R. massiliae* (MH532237)
		ompARmasRs4	1	*Rh. sanguineus* s.l.	Beja	MW026195	99.8% *R. massiliae* (MH532237)
		ompARmasRt1	16	*Rh. turanicus*	Beja	MN311231	100% *R. massiliae* (MH532237)
		ompARmasRt2	4	*Rh. turanicus*	Beja	MW026200	100% *R. massiliae* (KJ663747)
		ompARmasRt3	5	*Rh. turanicus*	Beja	MW026204	99.8% *R. massiliae* (MH532237)
		ompARmasRt4	2	*Rh. turanicus*	Beja	MW026209	99.8% *R. massiliae* (MH532237)
		ompARmasRt5	2	*Rh. turanicus*	Beja	MW026211	99.6% *R. massiliae* (MH532237)
		ompARmasRt6	1	*Rh. turanicus*	Beja	MW026213	99.8% *R. massiliae* (MH532237)
		ompARmasRt7	1	*Rh. turanicus*	Bizerte	MW026214	99.6% *R. massiliae* (KJ663747)
*gltA*	*R. massiliae*	gltARmasRt1	25	*Rh. turanicus*	Bizerte and Beja	MW026215	100% *R. massiliae* (KJ663740)

*R. sanguineus s.l., Rhipicephalus sanguineus sensu lato; R. turanicus, Rhipicephalus turanicus*.

a *Number of sequenced Rickettsia positive samples*.

b *Geographical location*.

c *GenBank accession number*.

**Figure 2:**
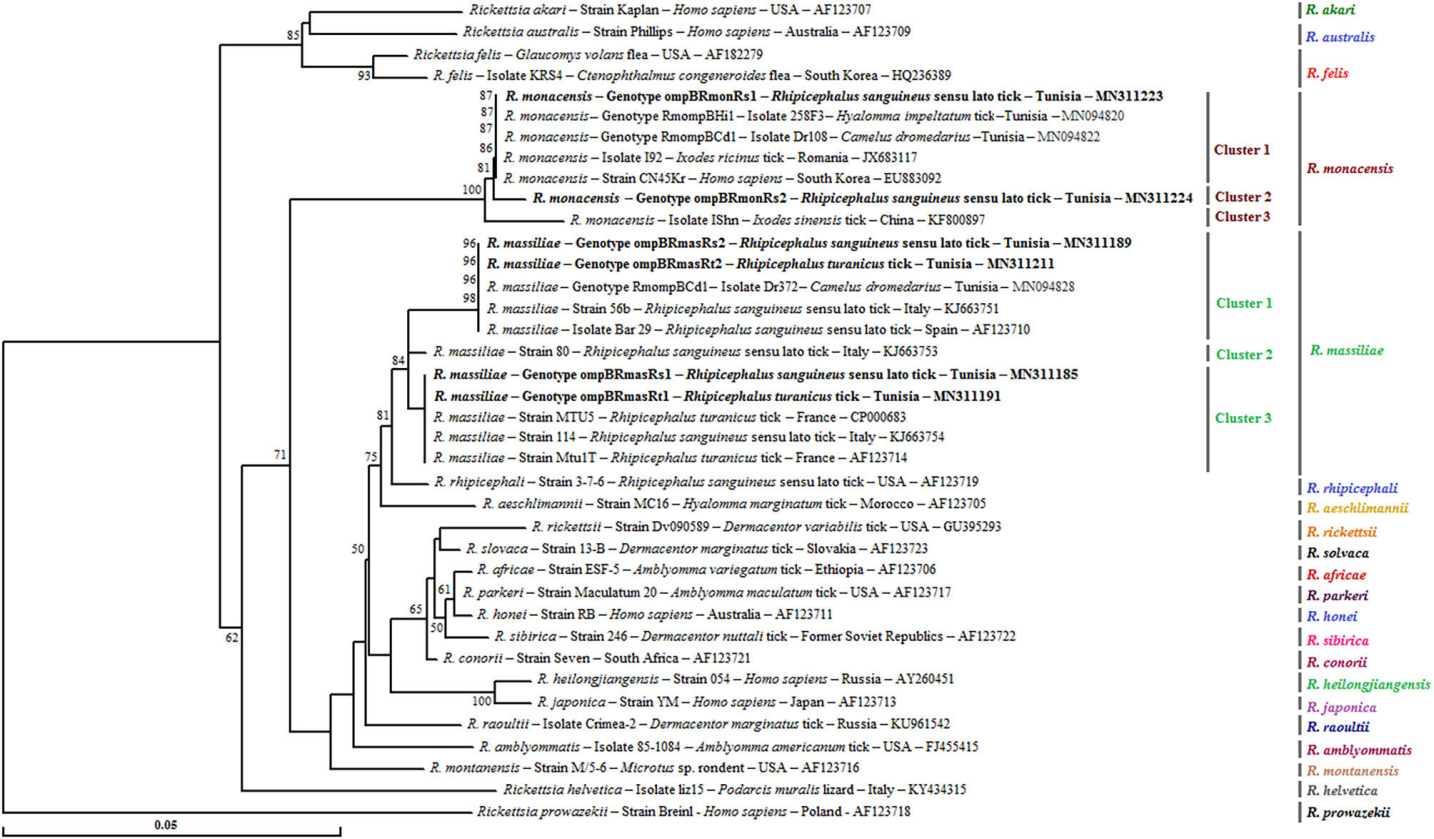
Neighbor-joining tree based on the alignment of partial *ompB* sequences (382 bp) of *Rickettsia* spp. obtained in this study with selected sequences representative of the *Rickettsia* genus. Numbers over the branches indicate the percentage of replicated trees in which the associated taxa clustered together in the bootstrap test (1,000 replicates, only percentages >50% were represented). The six partial *ompB* sequences representative of different *Rickettsia* spp. genotypes obtained in this study are indicated in bold. The host or vector, the genotype, strain or isolate name, the country of origin, and the GenBank accession number are indicated. One *R. prowazekii ompB* partial sequence was added as an outgroup.

**Figure 3:**
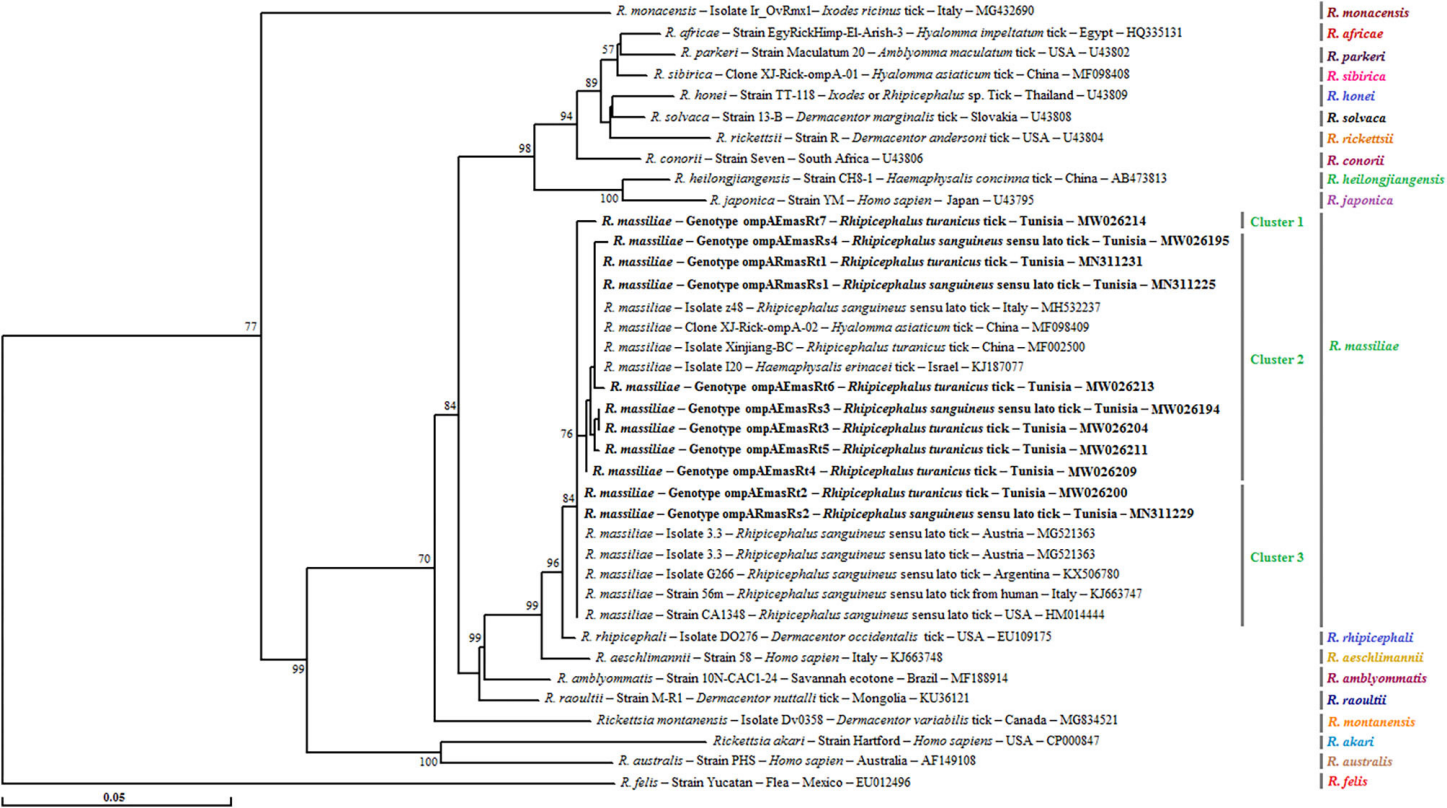
Phylogenetic tree of *Rickettsia* species inferred with partial sequences (490 bp) of the *ompA* gene using the neighbor-joining method showing the novel obtained sequences (*n* = 11) from Tunisian small ruminant ticks. Bootstrap values (1,000 replicates) are indicated in each node (only percentages >50% are shown). The 11 genotypes of *Rickettsia* spp. obtained in the present study are indicated in bold. The host or vector, the genotype, strain or isolate name, the country of origin, and the GenBank accession number are represented. One *R. felis ompA* partial sequence was added as an outgroup.

**Figure 4:**
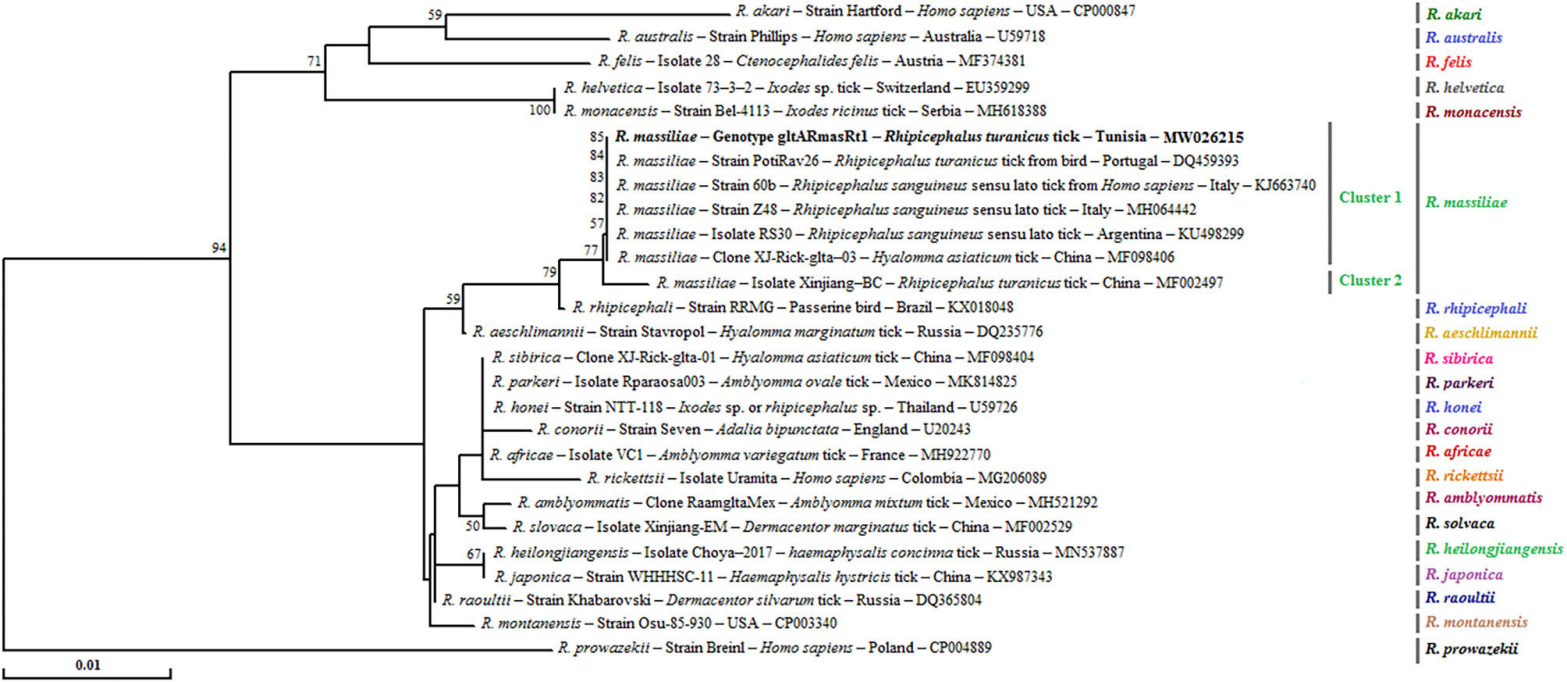
Phylogenetical relationships based on nucleotide multiple alignments of partial *Rickettsia* spp. *gltA* sequences (341 bp). Numbers over the branches indicate the percentage of replicated trees in which the associated taxa clustered together in the bootstrap test (1,000 replicates, only percentages >50% were represented). The only *R. massiliae gltA* genotype revealed in this study from 25 positive samples is represented in bold. The host or vector, the genotype, sequence type, strain or isolate name, the country of origin, and the GenBank accession number are indicated. One *R. prowazekii gltA* partial sequence was added as an outgroup.

*All information about the GenBank accession numbers represented in the Blast analysis is shown on the phylogenetic trees presented in Figures 2–4. Genotypes ompBRmasRs1, ompBRmasRs2, ompBRmasRt1, and ompBRmasRt2 were also represented by GenBank accession numbers MN311186–MN311188, MN311190, MN311192–MN311210, and MN311212–MN311222, respectively. Genotypes ompARmasRs1, ompARmasRs2, ompARmasRt1, ompARmasRt2, ompARmasRt3, ompARmasRt4, and ompARmasRt5 were also represented by GenBank accession numbers MN311226–MN311228, MW026192, MW026193, MN311230, MN311232–MN311242, MW026196–MW026199, MW026201–MW026203, MW026206–MW026208, MW026210, and MW026212, respectively. Genotype gltARmasRt1 was also represented by GenBank accession numbers MW026216–MW026239*.

#### *Rickettsia* spp. *ompB* Genotypes

*Rickettsia* infection was confirmed by sequencing of 382-bp *ompB* fragments from randomly selected 32 *Rh. turanicus*- and eight *Rh. sanguineus* s.l. *Rickettsia*-positive samples (Tables 3, 4). Alignment of these sequences revealed two *R. massiliae* genotypes from *Rh. sanguineus* s.l. (ompBRmasRs1 and ompBRmasRs2; GenBank accession numbers MN311185 and MN311189, respectively) and two *R. massiliae* genotypes from *Rh. turanicus* ticks (ompBRmasRt1 and ompBRmasRt2; GenBank accession numbers MN311191 and MN311211, respectively) (Table 4). In addition, two *R. monacensis* genotypes from *Rh. sanguineus* s.l. (ompBRmonRs1 and ompBRmonRs2; GenBank Accession Numbers MN311223 and MN311224, respectively) were also recorded (Table 4).

A phylogenetic analysis based on the alignment of Tunisian genotypes with 31 *Rickettsia* spp. *ompB* sequences obtained from GenBank shows the assignment of revealed genotypes to *R. massiliae* and *R. monacensis* clusters. The *R. massiliae* cluster is formed by three subclusters supported by robustness node rates ≥ to 81% (Figure 2). Tunisian strains were assigned to the first and third subclusters. Genotypes ompBRmasRs2 and ompBRmasRt2 were assigned to the first subcluster and clustered with strains isolated from *H. impeltatum* infesting camels in Tunisia and from *Rh. sanguineus* s.l. ticks located in Mediterranean countries such as Italy and Spain (Figure 2). Genotypes ompBRmasRs1 and ompBRmasRt1 were assigned to the third subcluster and clustered with strains isolated from *Rh. sanguineus* s.l. and *Rh. turanicus* ticks originated from North-Mediterranean countries (Figure 2). The *R. monacensis* cluster is also formed by three subclusters supported by robustness rates of nodes ≥ to 81% (Figure 2). Genotypes ompBRmonRs1 and ompBRmonRs2 were assigned, respectively, to the first and second subclusters. Genotype ompBRmonRs1 was closely related to isolates found in Tunisian camels and their infesting *H. impeltatum* ticks, and strains infecting human and ticks from different countries (Figure 2).

#### *Rickettsia* spp. *ompA* Genotypes

By using the *ompA* partial sequence, the infection with *R. massiliae* was revealed by sequencing of 490 bp of the *ompA* gene from selected 31 *Rh. turanicus*- and 10 *Rh. sanguineus* s.l. *Rickettsia*-positive samples (Tables 3, 4). Alignment of these sequences confirmed the occurrence of four distinct genotypes from *Rh. sanguineus* s.l. ticks (ompARmasRs1 to ompARmasRs4; GenBank Accession Numbers MN311225, MN311229 MW026194, and MW026195, respectively) and seven genotypes from *Rh. turanicus* ticks (ompARmasRt1 to ompARmasRt7; GenBank Accession Numbers MN311231, MW026200, MW026204, MW026209, MW026211, MW026213, and MW026214, respectively) (Table 4).

For this gene, a phylogenetic tree based on the alignment of *ompA* partial sequences of *Rickettsia* spp. found in GenBank showed the presence of our sequences in the three subclusters that formed the *R. massiliae* cluster and supported by robustness node rates ≥ to 84% (Figure 3). Genotype ompARmasRt7 formed separately subcluster 1, and genotypes ompARmasRt2 and ompARmasRs2 were assigned to the last subcluster and clustered with strains isolated from *Rh. sanguineus* s.l. located in different worldwide countries such as Italy, Austria, Argentina, and the USA. The remaining genotypes were clustered together in the second subcluster with several isolates infecting ticks from China and European countries (Figure 3).

#### *Rickettsia* spp. *gltA* Genotypes

Sequencing of 341 bp of the *gltA* partial sequence obtained from 25 specimens of *Rh. turanicus*-positive to *Rickettsia* spp. confirmed the infection with only one genotype (gltARmasRt1, GenBank accession number KJ663740) of *R. massiliae* (Tables 3, 4). This revealed that the genotype was 100% identical to strain 60B infecting *Rh. sanguineus* s.l. tick collected from Italian human (GenBank Accession Number KJ663740) (Table 4).

Phylogenetic tree based on the *gltA* gene revealed that the gltARmasRt1 genotype clustered in the *R. massiliae* cluster especially in the first subcluster 1 with strains infecting *Rh. sanguineus* s.l. ticks from Italy and Argentina, *Hyalomma asiaticum* ticks from China, and *R. turanicus* tick specimens collected from birds in Portugal (Figure 4).

## Discussion

Data about the occurrence and the genetic diversity of *Rickettsia* species in ticks is limited in North African countries (30, 31), especially in Tunisia (6, 8, 9). In this report, adult ticks infesting small ruminants in northern Tunisia were examined and two species of *Rhipicephalus* genus (*R. turanicus* and *R. sanguineus* s.l.) were identified. This result is in agreement with other surveys which considered these two tick species as major ectoparasites of small ruminants in Tunisia (32, 33).

To our knowledge, we report here for the first time the detection of SFG *Rickettsia* DNA in ticks collected from small ruminants raised in the north of Tunisia. Although this study does not conclude on the competence of these potential vectors, given that these results do not suggest that the tick species mentioned in this report can serve as a competent vector for detected bacteria, this study made a contribution to the knowledge of the presence of SFG rickettsiae in Tunisia. In addition, present data showed the need to search these bacteria in animal hosts and to increase the investigated areas, the potentially incriminated risk factors, and the number of analyzed tick samples, including questing ticks and different life stages. All these information may facilitate future prevention against SFG *Rickettsial* diseases in the country.

Specifically, the detection of *Rickettsia* spp. DNA in *Rh. turanicus* (23.4%) and *Rh. sanguineus* s.l. (9.5%) provides evidence that these tick species may be among the main vectors of *Rickettsia* species in northern Tunisia. These results are consistent with those reported by Khrouf et al. (6) who suggested a possible incrimination of *Rhipicephalus* ticks infesting dogs and sheep in the transmission of *Rickettsia* species in central Tunisia. Furthermore, according to Psaroulaki et al. (34), *Rickettsia* spp. were detected in *Rhipicephalus* ticks collected from domestic animals in Greece. Additionally, Germanakis et al. (35) reported that *Rh. turanicus* has been implicated as a potential vector transmitting to humans several pathogens including *Rickettsia* species. In the Northwest of China, Wei et al. (36) suggested that *R. massiliae, R. aeschlimannii*, and *R. sibirica* variants co-circulate in *R. turanicus* ticks. This data was confirmed by another study conducted by Song et al. (37) in the same country that indicates the occurrence of several SFG rickettsiae in *Rh. turanicus* collected from several ruminants. *Rickettsia massiliae* DNA was previously found in the salivary glands, and saliva of *Rh. turanicus* and its specific antibodies were also detected in patient sera. This may suggest, firmly, that *Rh. turanicus* act as a potential vector and reservoir for this bacterium (38).

Furthermore, analysis of potential risk factors demonstrated three interesting facts related to geographic regions, potential tick vector species, and infested hosts. Firstly, the positive rates of SFG *Rickettsia* in ticks were significantly higher in Beja (39%) than in Bizerte (13.9%) governorate. This discrepancy in prevalence rates according to geographic regions could be mainly explained by the diversity and heterogeneity of livestock population especially in El Alia locality and differences in husbandry practices, farm organization, wildlife reservoir hosts, and/or abiotic factors like the air temperature and the relative humidity that significantly affect the distribution of potential tick vectors. In addition, the higher rate of *Rickettsia* spp. observed in the governorate of Beja exclusively represented by the locality of Amdoun may be partly explained by the abundant presence in this region of *I. ricinus* considered to be one of the most important vectors of rickettsiae around the world (9). The infection of *Rhipicephalus* ticks with *Rickettsia* species may therefore come from infected small ruminants earlier infested with *Rickettsia*-positive *I. ricinus* ticks during wet seasons (9). Secondary, the positive rate in *Rh. turanicus* ticks (23.4%) was significantly higher compared to *Rh. sanguineus* s.l. (9.5%). This result is in line with those presented by Ghafar et al. (39) indicating a higher prevalence of *R. massiliae* and *R. slovaca* infections in *Rh. turanicus* ticks from Pakistan compared to other tick species. Furthermore, risk factor analysis showed that ticks collected from goats (23.2%) were more infected with *Rickettsia* spp. than those infesting sheep (7.9%) which is consistent with the same result of Ghafar et al. (39) in Pakistan.

In this study, *R. massiliae* was detected in *Rh. turanicus* and *Rh. sanguineus* s.l., thus confirming its occurrence especially in the north of Tunisia. In our country, previous studies have reported the presence of *R. massiliae* in *Rh. sanguineus* s.l. ticks collected from sheep situated in the center (6) and more recently in camels located in the center and the south (8). Similarly, *R. massiliae* has been also identified in *Rh. turanicus* and *Rh. sanguineus* s.l. from Algeria, Italy, Cyprus, and Greece (15, 34, 40), in *Rh. sanguineus* s.l. ticks from Morocco (41, 42), Spain, and Italy (12, 43), and in *Rh. turanicus* ticks from China (36) and Pakistan (39).

In the present study, *R. monacensis* DNA was detected in *Rh. sanguineus* s.l. tick specimens removed from goats. These results consolidate previous data describing the presence of this bacterium in questing *I. ricinus* ticks (9), and in camels and their infesting *H. impeltatum* ticks (8). Besides, wide geographical distribution of this pathogen was noted particularly in the Mediterranean region (Italy and Spain) and from other countries like Costa Rica and Nicaragua (44–46). Interestingly, this species was identified as a zoonotic pathogen able to cause from moderate to severe illness in humans (19). The detection in Tunisia of *R. monacensis* DNA in *Rh. sanguineus* s.l. ticks collected from goats suggests that, even if the circulation in the environment is essentially maintained by *I. ricinus* ticks, there may be other species incriminated in the transmission of this bacterial species as suggested in other reports from several countries (19, 47). Our findings highlight the need of extensive studies in the *Rh. sanguineus* s.l. tick complex collected from small ruminants and other domestic animals principally dogs to assess and predict the potential risks for humans.

However, given the growing occurrence of novel *Rickettsia* species with unidentified pathogenicity, it will be essential to carry out supplementary genetic characterization of the revealed *Rickettsia* spp. by using a combination of genetic markers such as *ompA*, and *gltA*, in addition to the *ompB* gene. In the present study, phylogenetic trees based on the three gene fragments showed higher genetic diversity among the revealed *R. massiliae* isolates by using *ompA* and *ompB* genes compared to the *gltA* gene. This result is in line with those presented by Ereqat et al. (11) and Chisu et al. (48) investigating Palestinian and Sardinian ticks, respectively.

By analyzing *ompB* partial sequences, two genotypes (ompBRmasRs1 and ompBRmasRt1) infecting *Rh. turanicus* and *Rh. sanguineus* s.l. tick specimens were found similar to that isolated from *R. massiliae* strain MTU5 (CP000683) recovered from *Rh. turanicus* ticks collected on horses in Camargues, France (49), suggesting its potential spread in several Mediterranean countries. The remaining genotypes (ompBRmasRs2 and ompBRmasRt2) also infecting both tick species were found identical to *R. massiliae* Bar29 (AF123710) earlier identified in *Rh. sanguineus* s.l. ticks from Spain based on the same gene (50) and from Tunisia based on the 23S-5S intergenic spacer (6). Additionally, on the basis of the *ompA* phylogenetic tree, we found that *R. massiliae* isolated from *Rhipicephalus* ticks showed genetic divergence with novel genotypes, which indicates that these isolates infecting different tick species may come from various origins, hosts, and reservoirs. Thus, this finding needs to be further investigated.

Based on *ompB* phylogeny, low genetic diversity was observed among *R. monacensis* genotypes identified in this study. Indeed, one genotype (ompBRmonRs1) was found to be 100% similar to the corresponding sequence of *R. monacensis* strain CN45Kr (EU883092) infecting a patient from South Korea (51), revealing its widespread distributions and potential risk for human. Thus, for a more accurate classification of our revealed *R. monacensis* isolates, further testing and phylogenetic analysis with additional genes are needed since no sequences of the two other genes isolated from this *Rickettsia* species were obtained in this study.

Therefore, the observation of these two zoonotic *Rickettsia* species, *R. massiliae* and *R. monacensis*, in investigated regions indicates a possible threat to resident humans. Indeed, infected tick species can also infest various domesticated animals and therefore constitute a possible risk for transmission of SFG rickettsiae to humans (3). However, the pathogenicity of this bacterium to humans is not well-understood (48). Consequently, supplementary trials are needed to investigate the pathogenicity of the revealed *Rickettsia* species and whether found tick species can transmit these pathogens in humans.

## Conclusions

The present study confirms the occurrence of human-pathogenic *Rickettsia* species in *Rh. sanguineus* s.l. and *Rh. turanicus* ticks collected from small ruminants in Tunisia. Our findings expand knowledge on ticks collected from domestic animals and highlight the range of infectious agents that may be transmitted by ticks to humans and animals.

## Data Availability Statement

The datasets presented in this study can be found in online repositories. The names of the repository/repositories and accession number(s) can be found in the article/supplementary material.

## Ethics Statement

The animal study was reviewed and approved by The Ethics Committee of the National School of Veterinary Medicine of Sidi Thabet, University of Manouba. Written informed consent was obtained from the owners for the participation of their animals in this study.

## Author Contributions

HB, LM, and MB conceived the idea. HB and MD-J carried out the fieldwork. HB, RS, and SZ performed the experiments. HB and MB performed risk factor analysis, genotyping, and phylogenetic study. HB and MB wrote the manuscript and HB, RS, LM, and MB finalized it. All authors read and approved the final version.

## Conflict of Interest

The authors declare that the research was conducted in the absence of any commercial or financial relationships that could be construed as a potential conflict of interest.
